# Air quality assessment and pollution forecasting using artificial neural networks in Metropolitan Lima-Peru

**DOI:** 10.1038/s41598-021-03650-9

**Published:** 2021-12-20

**Authors:** Chardin Hoyos Cordova, Manuel Niño Lopez Portocarrero, Rodrigo Salas, Romina Torres, Paulo Canas Rodrigues, Javier Linkolk López-Gonzales

**Affiliations:** 1 E.P. Ingeniería Ambiental, Facultad de Ingeniería y Arquitectura, Universidad Peruana Unión, Lima, Peru; 2 Escuela de Ingeniería C. Biomédica, Universidad de Valparaíso, Valparaíso, Chile; 3 Engineering Faculty, Universidad Andres Bello, Viña del Mar, Chile; 4 Department of Statistics, Federal University of Bahia, Salvador, Brazil; 5 Instituto de Estadística, Universidad de Valparaíso, Valparaíso, Chile

**Keywords:** Environmental sciences, Environmental social sciences

## Abstract

The prediction of air pollution is of great importance in highly populated areas because it directly impacts both the management of the city’s economic activity and the health of its inhabitants. This work evaluates and predicts the Spatio-temporal behavior of air quality in Metropolitan Lima, Peru, using artificial neural networks. The conventional feedforward backpropagation known as Multilayer Perceptron (MLP) and the Recurrent Artificial Neural network known as Long Short-Term Memory networks (LSTM) were implemented for the hourly prediction of $\hbox {PM}_{10}$ based on the past values of this pollutant and three meteorological variables obtained from five monitoring stations. The models were validated using two schemes: The Hold-Out and the Blocked-Nested Cross-Validation (BNCV). The simulation results show that periods of moderate $\hbox {PM}_{10}$ concentration are predicted with high precision. Whereas, for periods of high contamination, the performance of both models, the MLP and LSTM, were diminished. On the other hand, the prediction performance improved slightly when the models were trained and validated with the BNCV scheme. The simulation results showed that the models obtained a good performance for the CDM, CRB, and SMP monitoring stations, characterized by a moderate to low level of contamination. However, the results show the difficulty of predicting this contaminant in those stations that present critical contamination episodes, such as ATE and HCH. In conclusion, the LSTM recurrent artificial neural networks with BNCV adapt more precisely to critical pollution episodes and have better predictability performance for this type of environmental data.

## Introduction

The World Health Organization (WHO) reported that air pollution causes 4.2 million premature deaths per year in cities and rural areas around the world^1^. The US Environmental Protection Agency^2^ mentions that one of the pollutants with the most significant negative impact on public health is particulate material with a diameter of less than ten $\mathrm {\mu m}$ ($\hbox {PM}_{10}$) because it can easily access the respiratory tract causing severe damage to health. For their part, Valdivia and Pacsi^3^ report that Metropolitan Lima (LIM) is vulnerable to high concentrations of $\hbox {PM}_{10}$, due to its accelerated industrial and economic growth, in addition to its large population, as it is home to 29% of the total Peruvian population^4^.

To mitigate the damage caused by $\hbox {PM}_{10}$ to public health, the WHO established concentration thresholds suitable to achieve a minimum adverse effect on health^5^. In various countries, several laws were issued to regulate $\hbox {PM}_{10}$ concentrations and air quality in general^6^, as established in Peru by the Ministry of the Environment^7^ and in, e.g., the United States by the Environmental Protection Agency (EPA)^8^.

In recent years, various forecasting methodologies have been adapted and developed to understand how pollutants behave in the air at the molecular level, simulating diffusion and dispersion patterns based on the size and type of the molecule. However, the results of the prediction tend to achieve a somehow low precision^9,10^. Examples of such models are the Community Multiscale Air Quality model and the Weather Research and Forecasting model coupled with Chemistry developed in Chen et al.^11^ and Saide et al.^12^, respectively, which are used to forecast air quality in urban areas. On the other hand, some methods tend to be more appropriate to model and forecast air quality because they use statistical modeling techniques, such as Artificial Neural Networks (ANNs). These models have been widely used to forecast time series and applied to environmental data such as particulate matter in different countries^13,14^.

Several studies have been focusing on applying recurrent neural networks to forecast air quality in large cities. For instance, Guarnaccia et al.^15^ reported that predicting air quality with high accuracy can be problematic. This issue is becoming increasingly important because it is a tool capable of providing complete information for helping to prevent critical pollution episodes and reduce human exposure to these contaminants^13,16,17^. However, there is a limited number of studies in the context of Lima, Peru, which is one of the cities with the highest pollution levels in South America^18–20^. For instance, Herrera and Trinidad^21^ used neural networks to predict $\hbox {PM}_{10}$ in the Carabayllo district - Lima, with a good forecasting performance. Salas et al.^22^ developed a NARX model using artificial neural networks to predict the $\hbox {PM}_{10}$ pollutant in Santiago, Chile. Athira et al.^23^ aimed at forecasting $\hbox {PM}_{10}$ three days ahead and at comparing the performance of the standard LSTM, GRU, and RNN models, concluding that all three models showed good performance for out-of-sample forecasting.

Lima is considered to be one of the most polluted cities in Latin America in terms of $\hbox {PM}_{10}$. In this sense, the need for sophisticated environmental management instruments arises, aiming at making predictions with greater precision using cutting-edge methodologies, such as deep learning algorithms, which support decision-making to establish mitigation and prevention policies. In addition, it allows the population to avoid being exposed to high concentrations of $\hbox {PM}_{10}$. For this reason, this study aims to assess the air quality of Lima, to understand its behavior, and the possible causes and factors that favor pollution. Subsequently, we applied the Multilayer Perceptron (MLP) and the Long Short-Term Memory (LSTM) models to forecast $\hbox {PM}_{10}$ concentrations, where the models were evaluated under two validation schemes: the Hold-out (HO) and the Block Nested Cross-Validation (BNCV). Our contributions are summarized below:

- In this study, we have implemented artificial neural networks to model time series data collected from five meteorological and air quality monitoring stations from Lima, Peru. The monitoring stations are ATE, Campo de Marte (CDM), Carabayllo (CRB), Huachipa (HCH) and San Martin de Porres (SMP). We have investigated the geographical and meteorological divergence of the forecast results from the five air quality monitoring areas in LIM using data collected from two years.
- The proposed time series forecasting model based on the MLP and LSTM neural networks efficiently predicted one-hour-ahead $\hbox {PM}_{10}$ concentrations. The prediction performances between the five stations were compared. According to the literature review, this study is the first to use deep learning algorithms to predict air quality ($\hbox {PM}_{10}$) in LIM.
- We have focused the study in LIM because its air pollution has worsened in recent years. The main reason for this change is that population growth has been unsustainable, and high industrial activity and the accelerated growth of the automobile fleet have increased. These factors make it challenging to predict $\hbox {PM}_{10}$ pollution concentrations.

The remainder of the paper is structured as follows: Section “Materials and methods” presents the developed methodology based on an exploratory study described in two phases. In Section 3, we present the main results and their discussion. Finally, in Section 4, we provide the main conclusions and give some future works.

## Materials and methods

**Figure 1:**
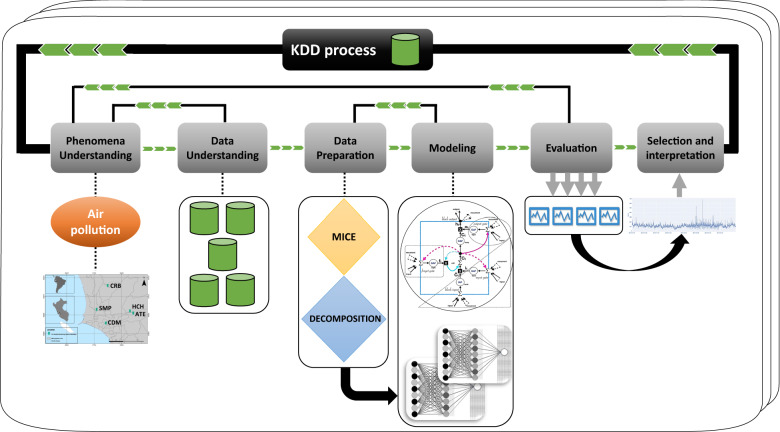
Knowledge Discovery from Databases (KDD) methodology used for Air Quality Assessment and Pollution Forecasting.

In this work, we follow the Knowledge Discovery from Databases (KDD) methodology to obtain relevant information for air quality management decision-making. The main goal of the KDD is to extract implicit, previously unknown, and potentially helpful information^24^ from raw data stored in databases. Therefore, the resulting models can predict, e.g., one-hour ahead, the air quality and support the city’s management decision-making (see Fig. 1).

The KDD methodology has the following stages: (a) Phenomena Understanding; (b) Data Understanding; (c) Data Preparation; (d) Modeling; (e) Evaluation; and, (d) Selection/Interpretation. In the following subsections, we explain each stage of the process.

### Phenomena Understanding

In this first stage, we contextualize the contamination phenomenon concerning the $\hbox {PM}_{10}$ concentrations in the five Lima monitoring stations. The main focus is to predict air pollution to support decision-making related to establishing pollution mitigation policies. For this, we use both MLP and LSTM as computational statistical methods for $\hbox {PM}_{10}$ prediction.

Lima is the capital of the Republic of Peru. It is located in the center of the western side of the South American continent in the $77^{\circ }$ W and $12^{\circ }$ S and, together with its neighbor, the constitutional province of Callao, form a populated and extensive metropolis with 10,628,470 inhabitants and an area of $2819.3\,\hbox {km}^2$^25,26^.

The average relative humidity (temperature) in the summer (December–March) ranges from 65–68% (24 °C–26 °C) in the mornings, while at night the values fluctuate between 87–90% (18 °C–20 °C). In the winter (June–September), the average daytime relative humidity (temperature) ranges between 85–87% (18 °C–19 °C) and at night it fluctuates between 90–92% (18 °C–19 °C). The average annual precipitation is 10 mm. On the other hand, the average altitudes reached by the thermal inversion in summer and winter are approximately 500 and 1500 m above sea level, respectively^27,28^.

**Figure 2:**
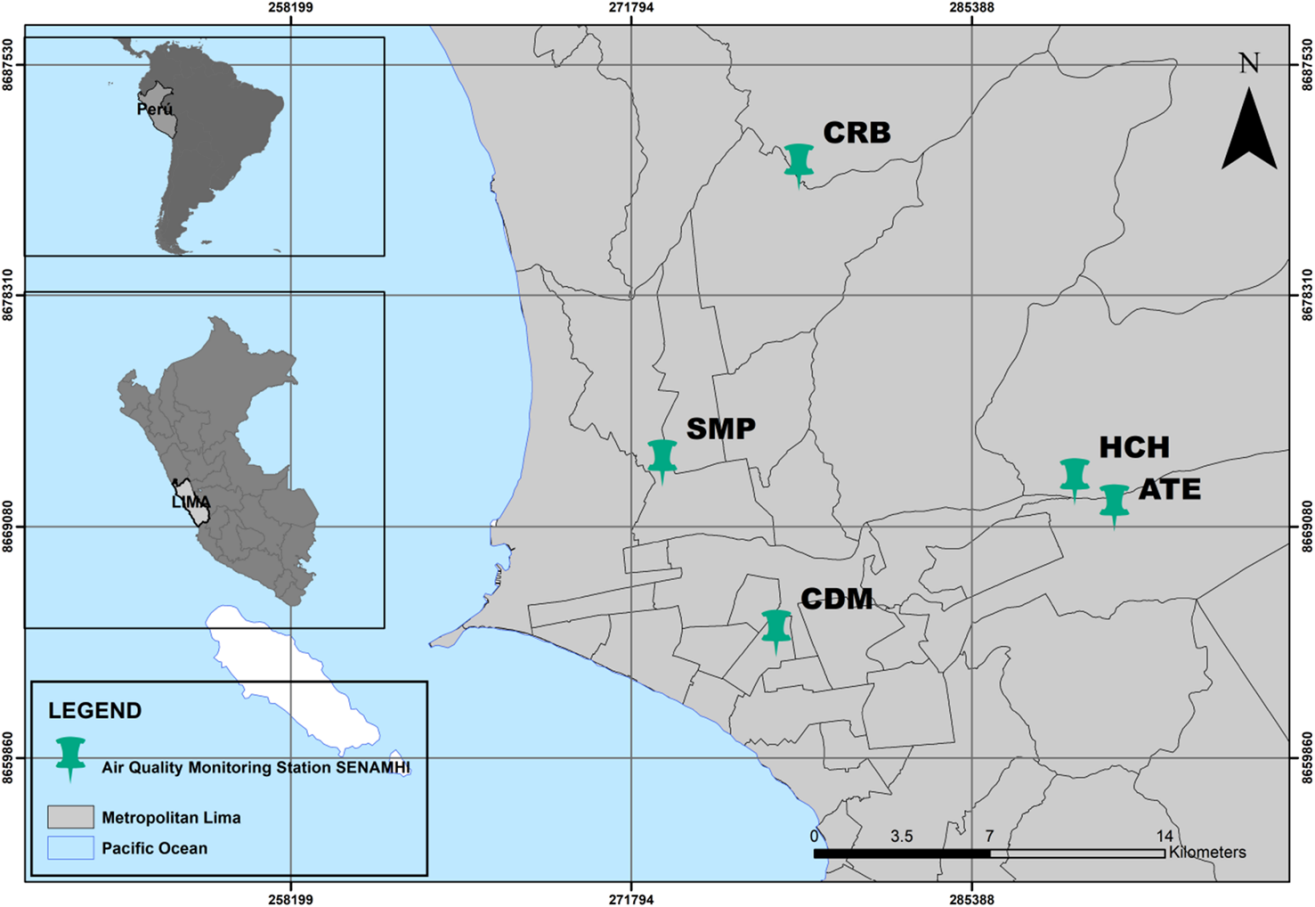
Map with the study area and the locations of the Lima air quality monitoring stations: ATE, Campo de Marte (CDM), Carabayllo (CRB), Huachipa (HCH) and San Martin de Porres (SMP).

**Table 1:** Pollutant and weather variables used in this study, and their units of measurement.

Variable	Unit of measurement
$\hbox {PM}_{10}$	$\mu \hbox {g}/\hbox {m}^{3}$
Temperature	$^{\circ }\hbox {C}$
Relative humidity	%
Wind speed	m/s
Wind direction	Degrees ($^{\circ }$)

### Data understanding

Lima has ten air quality monitoring stations located in the constitutional province of Callao and the north, south, east, and center of Lima. The data used comprise hourly observations from January 1st, 2017, to December 31st, 2018, and includes three meteorological variables and the concentration of particulate matter $\hbox {PM}_{10}$. Where the latter is considered to be an agent that, when released into the environment, causes damage to ecosystems and living beings^29,30^. For this study, the hourly data, recorded at five air quality monitoring stations (see Fig. 2), which are managed by the National Service of Meteorology and Hydrology of Peru (SENAMHI), was considered. Table 1 shows the considered variables and their units of measurement.

When considering environmental data, such as $\hbox {PM}_{10}$ concentrations, from different locations, preliminary spatio-temporal visualization studies are of great use to better understand the behavior of the meteorological variables, the topography of the area, and the pollutants^31^.

### Data preparation

This stage is very relevant because it precedes the modeling stage. The preparation of the data had various stages. First, we address the problem of missing data. The treatment was performed with the MICE library. This library performs multiple imputations using the Fully Conditional Specification^32^ and requires a specification of a separate univariate imputation method for each incomplete variable. In this context, predictive mean matching, a versatile semiparametric method focusing on continuous data, was used, which allows the imputed values to match one of the observed values for each variable. The data imputation was performed for each of the five stations with a percentage of missing data below 25%.

The data from the monitoring stations consist of a sequence of observed values $\{x_t\}$ recorded at specific times *t*. In this case, the time series is collected at hourly intervals. After the data imputation, we proceed to normalize all the observations in the range [0,1] as follows:

1 $$\begin{aligned} X_{t} = \frac{x_{t} - \min \{x_t\}}{\max \{x_t\} - \min \{x_t\}} \end{aligned}$$

Moreover, the time series is decomposed into the trend, seasonality, and the irregular components following an additive model (the cyclic component is omitted in this work):

2 $$\begin{aligned} X_t = Trend_t + Cyclic_t + Seasonal_t + Irregular_t \end{aligned}$$

The trend component $Trend_t$ at time *t* reflects the long-term progression of the series that could be linear or non-linear. The seasonal component $Seasonal_t$ at time *t*, reflects the seasonal variation. The irregular component $Irregular_t$ (or “noise”) at time *t* describes the random and irregular influences. In some cases, the time series has a cyclic component $Cyclic_t$ that reflects the repeated but non-periodic fluctuations. The main idea of applying this decomposition is to obtain the deterministic and the random components, where a forecasting model is obtained using the deterministic part^33,34^. In this article, we have used the method implemented in Statmodels for Python^35^, where a centered moving average filter is applied to the time series.

### Modeling using artificial neural networks

Artificial Neural Networks have received a great deal of attention in engineering and science. Inspired by the study of brain architecture, ANNs represent a class of non-linear models capable of learning from data^36^. The essential features of an ANN are the basic processing elements referred to as neurons or nodes, the network architecture describing the connections between nodes, and the training algorithm used to estimate values of the network parameters.

Researchers see ANNs as either highly parameterized models, or semiparametric structures^36^. ANNs can be considered as hypotheses of the parametric form $h(\cdot ;{{\mathbf {w}}})$, where the hypothesis *h* is indexed by the vector of parameters ${\mathbf {w}}$. The learning process consists of estimating the value of the vector of parameters ${\mathbf {w}}$ to adapt the learner *h* to perform a particular task.

Machine Learning and Deep learning methods have been successfully applied for time series forecasting^37–42^. For instance, recurrent artificial neural networks (RNNs) are dynamic models frequently used for processing sequences of real data step by step, predicting what comes next. They are applied in many domains, such as the prediction of pollutants^43^. It is known that when there are long-term dependencies in the data, RNNs are challenging to train, which leads to the development of models such as the LSTM that have been successfully applied in time series forecasting^44^.

**Figure 3:**
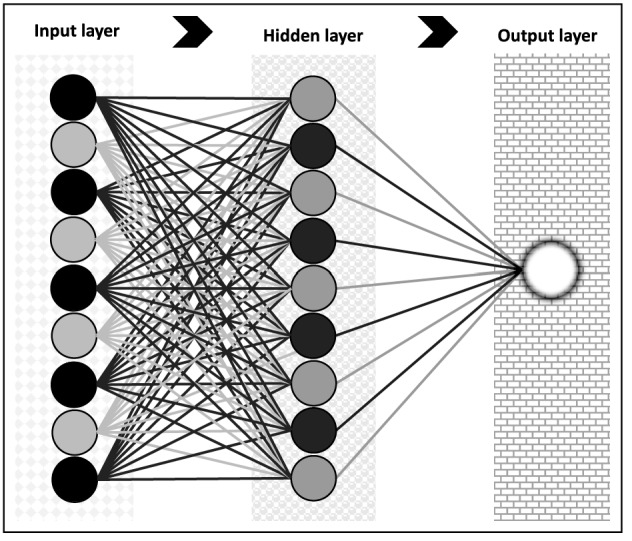
Schematic of the architecture of the Multilayer Perceptron. The figure shows three layers of neurons: input, hidden and output layers.

The Multilayer Perceptron model consists of a set of elementary processing elements called neurons^36,45–48^. These units are organized in architecture with three layers: input, hidden, and output. The neurons corresponding to one layer are linked to the neurons of the subsequent layer. Figure 3 illustrates the architecture of this artificial neural network with one hidden layer. The non-linear function ${\mathbf {g}}({\mathbf {x}},{\mathbf {w}})$ represents the output of the model, where ${\mathbf {x}}$ is the input signal and ${\mathbf {w}}$ being its parameter vector. For a three-layer FANN (one hidden layer), the *k*-th output computation is given by the following equation

3 $$\begin{aligned} g_k({\mathbf {x}},{\mathbf {w}})=f_2\left( \sum _{j=1}^{\lambda }w_{kj}^{[2]}f_1 \left( \sum _{i=1}^{d} w_{ji}^{[1]}x_i+w_{j0}^{[1]}\right) +w_{k0}^{[2]}\right) \end{aligned}$$

where $\lambda$ is the number of hidden neurons. An important factor in the specification of neural models is the activation function’s choice. These can be any non-linear functions as long as they are continuous, bounded, and differentiable. The transfer function of the hidden neurons $f_1(\cdot )$ should be nonlinear while for the output neurons the function $f_2(\cdot )$ could be a linear function or nonlinear functions. One of the most used functions is the sigmoid:

4 $$\begin{aligned} f(z)=\frac{1}{1+\exp (-z)} \end{aligned}$$

The MLP operates as follows. The input layer neurons receive the input signal; these neurons propagate the signal to the first hidden layer and do not make any processing. The first hidden layer processes the signal and transfers it to the subsequent layer; the second hidden layer propagates the signal to the third, and so on. When the signal is received and processed by the output layer, it generates the response.

The Long Short-Term Memory networks model is a type of RNN, having as its primary strength the ability to learn long-term dependencies and being a solution for long time series intervals^20,49^. In such a model, memory blocks replace the neurons in the hidden layer of the standard RNN^50^. The memory block consists of three gates that control the system’s state: Input, forget, and output gates. First, the input gate determines how much information will be added to the cell. Second, the forget gate controls the information lost in the cells. Lastly, the output gate performs the function of determining the final output value based on the input and memory of the cell^51,52^.

5 $$\begin{aligned} f_{t}= & {} \sigma \left( W_{f} \cdot \left[ h_{t-1}, x_{t}\right] +b_{f}\right) \end{aligned}$$

6 $$\begin{aligned} i_{t}= & {} \sigma \left( W_{i} \cdot \left[ h_{t-1}, x_{t}\right] +b_{i}\right) \end{aligned}$$

7 $$\begin{aligned} {\tilde{C}}_{t}= & {} \tanh \left( W_{{\tilde{C}}} \cdot \left[ h_{t-1}, x_{t}\right] +b_{{\tilde{C}}}\right) \end{aligned}$$

8 $$\begin{aligned} C_{t}= & {} \left( {f}_{t} \cdot C_{t-1}\right) + \left( i_{t} \cdot {\tilde{C}}_{t}\right) \end{aligned}$$

9 $$\begin{aligned} o_{t}= & {} \sigma \left( W_{o}\left[ h_{t-1}, x_{t}\right] +b_{o}\right) \end{aligned}$$

10 $$\begin{aligned} {{h}_{t}}= & {} {{o}_{t}}\cdot tanh({{C}_{t}}) \end{aligned}$$

**Figure 4:**
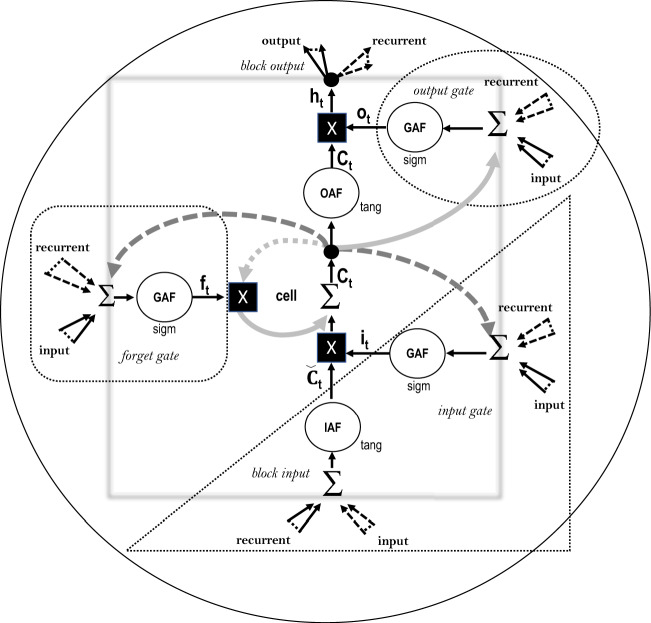
Model of one block of the LSTM. The block is composed of the input gate, forget gate and output gate.

Figure 4 shows the LSTM model block, with the output and input blocks, which consists of three gates. At each step, an LSTM maintains a hidden vector *h* and a memory vector *o* responsible for controlling status updates and outputs.

The first step is to decide what information will not be considered in the status cell. This decision is made by the forget gate, which uses a hyperbolic tangent activation function (IAF). $f_{t}$ represents the output of the forget gate, which can be calculated using equation (5). This gate considers the concatenation of the vectors $h_{t-1}$ and $x_t$. It generates a number between 0 and 1 for each number in the state cell $C_{t-1}$, where $W_f$ and $b_f$ are the weight matrices and the bias vector parameters, respectively. Both must be learned during training and are stored in the vector $f_t$. If one of the values of this vector is equal to or close to zero, then the LSTM will eliminate that information. On the other hand, if it reaches values equal to or close to 1, this information will be maintained and reach the status cell.

The next step is to decide what new information to store in the status cell. This is done by the input gate, linked to a sigmoid activation function (GAF), and with an output for that gate ($i_{t}$), all this is calculated by the equation (6, 7). In addition, for the input block, the hyperbolic tangent activation function (IAF) is used. First, the vectors $h_{t-1}$ and $x_t$ are concatenated. Being $W_i$ and $b_i$, the weight matrices and the bias vector parameters, respectively, must be learned during training; all this is stored in the vector $i_t$ called the input gate, which decides which values to update. Then a hyperbolic tangent function creates a vector of new candidate values, ${\tilde{C}}_{t}$, involving the vectors $h_{t-1}$ and $x_t$. In the next step, these values are filtered by multiplying point by point both vectors to create a status cell update. The previous cell, $C_{t-1}$ is updated to the new state of cell $C_t$ (equation 8).

In addition, the output gate, also linked with the GAF activation function and with an output of the output gate ($o_{t}$), for its calculation uses the equation (equation 9). Finally, $h_{t}$, expresses the new output of the model (equation 10). The current cell state is represented by $C_{t}$, while *W* is the weight vector o parameters of the model, and *b* is the bias of the model.

### Model evaluation

To evaluate the forecast ability of the models, the performance metrics given below were used (see^53,54^). In what follows, we will consider: $y_{i}$, $i=1,\ldots ,n$, are the target values; ${\hat{y}}_{i}$, $i=1,\ldots ,n$, are the model’s predictions; ${\bar{y}}_{i}$ is the mean of the target values; and *n* is the number of samples.

1. Mean Absolute Error: The average absolute difference between the target and the predicted values.
  11 $$\begin{aligned} \mathrm {MAE}=\frac{\sum _{i=1}^{n}\left| y_{i}-{\widehat{y}}_{i}\right| }{n} \end{aligned}$$
2. Root Mean Squared Error: The squared root of the average of the squared errors.
  12 $$\begin{aligned} \mathrm {RMSE}=\sqrt{\frac{\sum _{i=1}^{n}\left( y_{i} -{\widehat{y}}_{i}\right) ^{2}}{n}} \end{aligned}$$
3. Symmetric Mean Absolute Percentage Error: A measure of accuracy based on a percentage of relative errors.
  13 $$\begin{aligned} \mathrm {sMAPE}=\frac{100 \%}{n} \sum _{i=1}^{n} \frac{\left| y_{i}-{\widehat{y}}_{i}\right| }{\left| {\widehat{y}}_{i}\right| +\left| y_{i}\right| } \end{aligned}$$
4. Spearman’s rank correlation coefficient: A nonparametric correlation measure between the target and the prediction. Spearman’s correlation assesses monotonic relationships by using the rank of the variables.
  14 $$\begin{aligned} S = 1-\frac{6 \sum _{i=1}^{n} d_{i}^{2}}{n\left( n^{2}-1\right) } \end{aligned}$$
  where $d_{i} = rg(y_i) - rg({\hat{y}}_i)$ is the difference between the ranks of the targets $rg(y_i)$ and the predictions $rg({\hat{y}}_i)$.

### Model selection and interpretation

The model selection and interpretation is the final step in the KDD process and requires that the knowledge extracted from the previous step be applied to the specific domain of the $\hbox {PM}_{10}$ prediction in a visualized format. At this stage, in addition to selecting the model with the best precision in the prediction, it also drives the decision-making process based on the air quality assessment in Lima.

**Figure 5:**
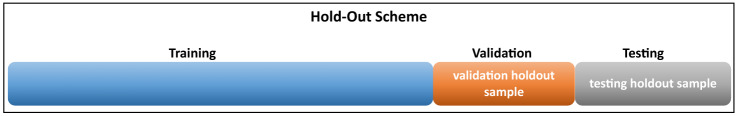
Hold-Out Scheme used for the validation of the models. The dataset is split into three sets: training, validation, and testing. The train set is the basis for training the model, and the test set is used to see how well the model performs in untrained $\hbox {PM}_{10}$ concentrations.

**Figure 6:**
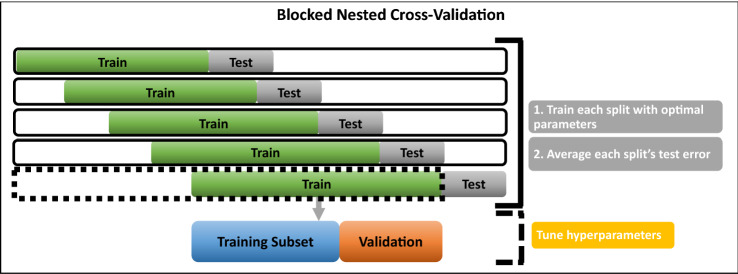
Blocked Nested Cross-Validation Scheme used for the validation of the models. The dataset is separated into three sets using a time-window of fixed size: training, validation, and testing. The last day is used for testing.

We have used two schemes for the validation: Hold-Out (HO) and Blocked Nested Cross-Validation (BNCV). On the one hand, HO has the conventional separation of the dataset in training, validation, and testing subsets (see Fig. 5). On the other hand, the BNCV is a fixed-size window that slides, and the model is retrained with all the data up to the current day (see Fig. 6).

## Results and discussion

### Air quality assessment in Metropolitan Lima-Peru

In this section, we report the results of the statistical analysis of air pollution in LIM.

#### Statistical analysis of the concentration of $\hbox {PM}_{10}$

**Figure 7:**
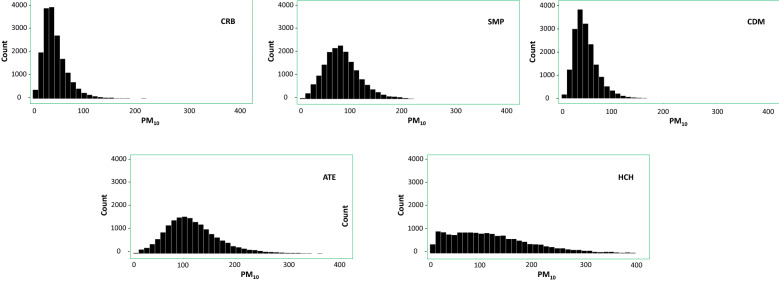
$\hbox {PM}_{10}$ Histograms for each of the five monitoring stations, respectively CRB, SMP, CDM, ATE, and HCH.

Table 2 shows the descriptive analysis of the data from the five monitoring stations focused in the $\hbox {PM}_{10}$, between 01-01-2017 and 31-12- 2018. Additionally, the histogram (see Fig. 7) is reported to show the behavior of the pollutant in every season. In the probability distribution, it is observed that they are skewed to the right, which indicates the existence of critical episodes of contamination, being the HCH station the one with the highest incidence, with an average of $130.03 \pm 91.68$ $\mu$g/$\hbox {m}^{3}$. This value exceeds that standardized by the Peruvian norm^7^, and shows relevant fluctuations and high dispersion of pollutants (8404.34 $\mu$g/$\hbox {m}^{3}$) that cause a high standard deviation. The stations HCH and ATE register higher concentration levels. The order of the stations from the lowest to the highest levels of the mean of $\hbox {PM}_{10}$ is as follows: **CRB**; **CDM**; **SMP**; **ATE**; **HCH**. Similar behaviour was found in other studies^31,55^. Encalada et al.^31^ carried out a study of visualization of $\hbox {PM}_{10}$ concentrations in Lima using the same data, where similar behavior patterns of $\hbox {PM}_{10}$ concentrations are shown in the five stations. In addition, all the stations surpass the $\hbox {PM}_{10}$ limits established by the WHO. Moreover, four of the five stations (except CRB) exceed the utmost limits of the annual arithmetic mean of $\hbox {PM}_{10}$ proposed in the Quality Standards Environmental (ECA) in Peru.

**Table 2:** Descriptive statistics for the five $\hbox {PM}_{10}$ monitoring stations.

SM	Minimun	Maximun	1st Qu.	3rd Qu.	Median	Mean ± DS	Variance	Skewness	Kurtosis
CRB	5.44	488.02	31.49	58.45	198.31	$48.69 \pm 28.39$	806.03	3.24	22.27
SMP	7.77	426.80	61.95	105.10	142.50	$86.05 \pm 35.73$	1276.41	1.00	2.86
CDM	6.08	463.60	35.84	63.45	145.50	$52.30 \pm 24.61$	605.54	2.30	18.25
ATE	6.41	931.00	82.90	148.00	421.90	$121.56 \pm 60.30$	3635.75	2.08	11.07
HCH	5.21	974.00	62.10	176.50	138.40	$130.03 \pm 91.68$	8404.34	1.53	4.89

#### Analysis of the correlations with the meteorological variables

**Figure 8:**
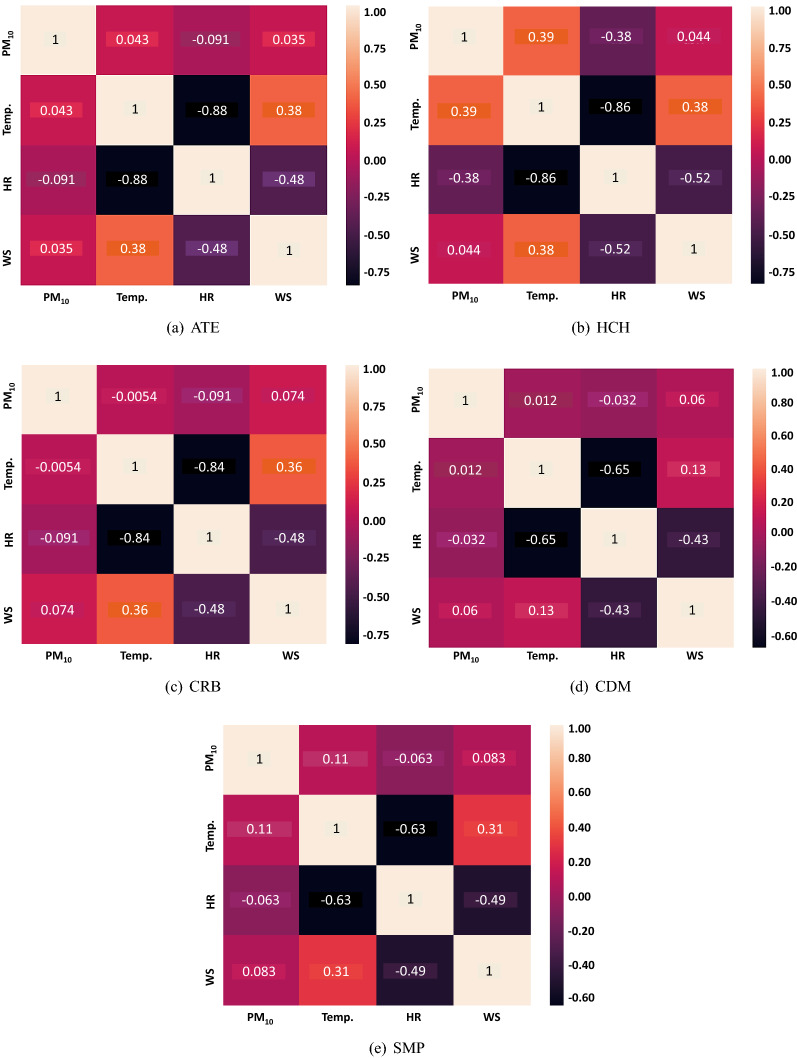
Correlation matrices between the meteorological variables and the $\hbox {PM}_{10}$ for each monitoring station.

A significant correlation between $\hbox {PM}_{10}$ and the meteorological variables was observed in the station HCH, which is the area with the highest $\hbox {PM}_{10}$ concentration. Factors such as dust, population / area ratio and weather conditions have a predominant effect on $\hbox {PM}_{10}$ concentration^56^. Figure 8 shows that there is a moderate positive correlation (0.39) between temperature and $\hbox {PM}_{10}$ and a moderate negative correlation (-0.38) between relative humidity and $\hbox {PM}_{10}$. This is due to the meteorological patterns that occur in the study area. According to Silva et al.^57^ between the years 1992 and 2014, the base of thermal inversions in Lima ranged between 0.6 and 0.9 kilometers from June to November and between 0.1 and 0.6 kilometers from December to May, having a minimum average of 0.13 kilometers in March, which coincides with the season that presents critical episodes of $\hbox {PM}_{10}$ concentrations.

**Figure 9:**
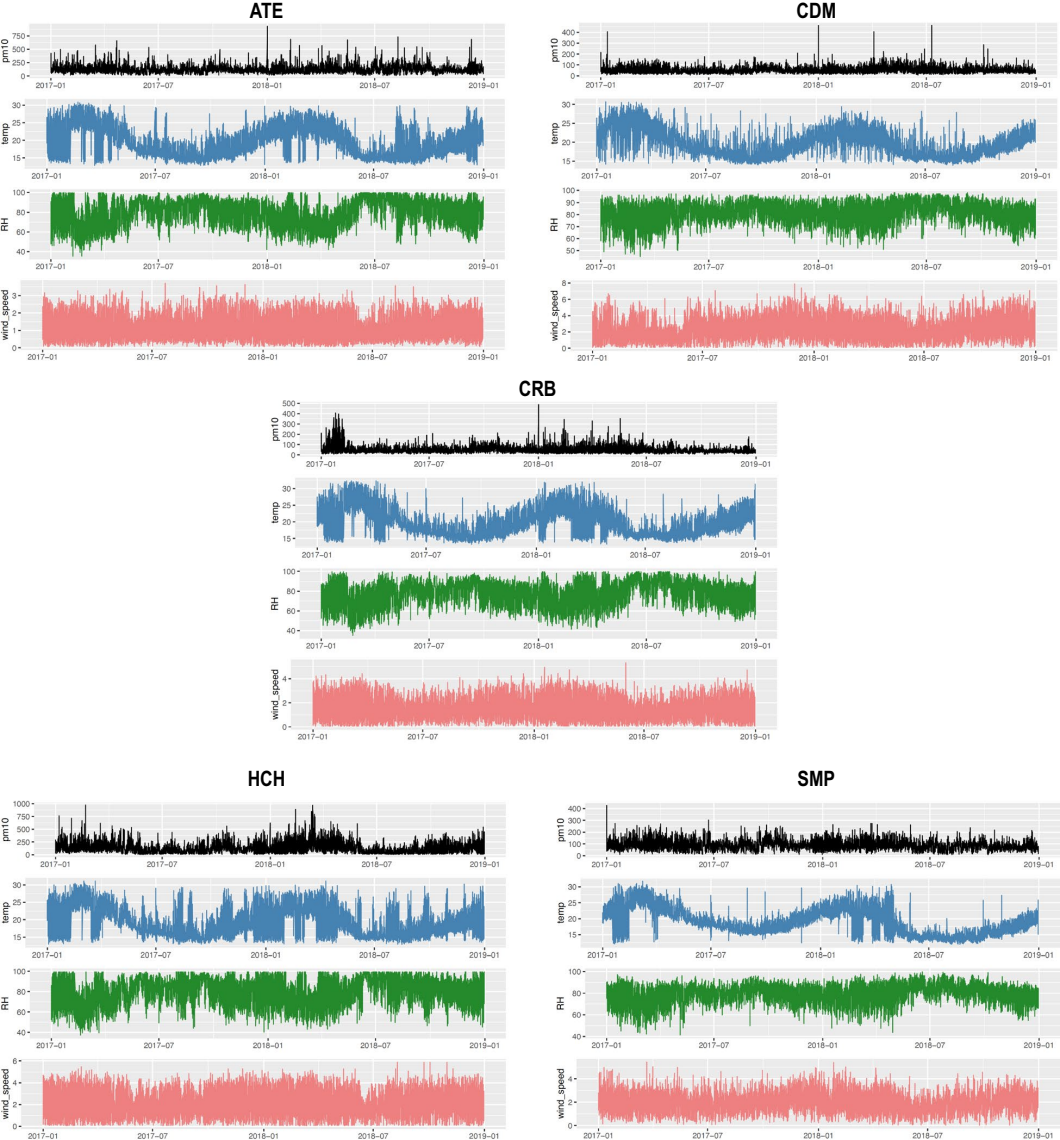
Time series of all variables, $\hbox {PM}_{10}$, temperature, relative humidity and wind speed, in each monitoring station, ATE, CDM, CRB, HCH and ATE, respectively.

The thermal inversion in the summer months reduces the dispersion of atmospheric pollutants because the density of the stratiform clouds decreases. Consequently, solar radiation leads to an increase in temperature and to a reduction in relative humidity. The latter results in a turbulent process causing the resuspension of coarse particles as $\hbox {PM}_{10}$^25^. High temperatures increase the photochemical activity that causes the decomposition of matter and, consequently, the increase of $\hbox {PM}_{10}$^58–60^. On the other hand, stratiform cloudiness increases in winter, as does relative humidity, that accompanied by drizzles in that season, help to significantly decrease the temperature and $\hbox {PM}_{10}$ concentrations due to wet deposition typical of the season^28^. The above explains the high negative correlation observed between temperature and relative humidity in the five monitoring stations (see Fig. 8), which is a normal phenomenon because the relative humidity directly depends on temperature and pressure to determine the capacity of the air in the intake of water vapor^61^. For this reason, the higher the temperature, the lower the relative humidity, as shown in Fig. 9.

#### Influence of wind direction and speed on $\hbox {PM}_{10}$ concentrations

The stations located in the highest area (eastern part) of the city have the highest concentration of $\hbox {PM}_{10}$. Contrary to the above, the stations located in the lowest area have a lower concentration of $\hbox {PM}_{10}$. This trend is due to the entry direction of persistent local winds from the coast to the south-southwest, which causes that pollutants such as $\hbox {PM}_{10}$ be transferred to the northeast and east areas of the city, making them in critical places of contamination by particulate matter^28,31^.

Although there is no significant correlation between wind speed and $\hbox {PM}_{10}$, this parameter has meteorological influence on the dispersion, resuspension, and horizontal transport of pollutants, provided that there are strong air currents (winds)^61–63^, which is not the case of the present study because the highest frequencies of wind speeds are between 0 – 3.10 m/s^31^.

The wind speed has a meteorological influence on the dispersion, suspension, and horizontal transport of pollutants provided that there are strong air currents (winds)^61–63^. However, this is not the case of the present study because the highest frequencies of wind speeds are between 0 and 3.10 m/s^31^, meaning that there is no significant correlation between wind speed and $\hbox {PM}_{10}$.

#### Critical episodes of $\hbox {PM}_{10}$ contamination at the HCH station

The station with the highest average $\hbox {PM}_{10}$ concentration between 2017 and 2018 is HCH (see Table 2). This area has the characteristic of high vehicular traffic compared to the rest of the stations considered. The Ramiro Prialé highway that crosses HCH and is the most used to access the central road connects the center and the east of the Peruvian territory, turning it into high traffic congestion. Moreover, 2,462,321 vehicles were circulating in Lima^64^ in 2017, and according to the National Institute of Statistics and Informatics (INEI), the vehicle fleet in Peru grew by 4.4% between 2017 and 2018^65^. The aforementioned explains the influence of high traffic vehicles in critical pollution episodes in HCH, which according to what is referred by Srishti et al.^66^, the traffic caused from vehicles contributes to about 21% of $\hbox {PM}_{10}$ of the pollution. In addition, it is associated with the wear of tires and brakes^64^.

Another particular feature of HCH compared to the other stations is the dilapidated, unpaved roads and the frequent inadequate disposal of land clearing on public roads by the population. These conditions generate a significant increase in dust, the main component of particulate matter, contributing to 54% of air pollution. The soil dust has a more significant impact in seasons or areas with little rainfall^66–68^. Furthermore, Lima is considered a city where it seldom rains and that only slight drizzles or wet haze breakouts from cloud-type clouds nimbostratus^69^.

In the surrounding area of HCH, there is also high industrial activity. Industrialization is directly associated with the increased generation of $\hbox {PM}_{10}$^69^. Concepción and Rodríguez^70^ note that both the industrial activity and the vehicle fleet are the leading causes of the generation of high concentrations of $\hbox {PM}_{10}$ in Lima, where the primary industries are brick kilns and non-metallic ore extraction. Moreover, it was evidenced that the HCH brick industries do not have the appropriate technology to mitigate air pollution and that in all their processes, high emission of particulate matter, from the movement of land to the burning of tires, plastics, or firewood in the ovens^71^. Added to all this, it is the lack of green areas in HCH, which facilitates the resuspension of $\hbox {PM}_{10}$.

#### Exploratory analysis on a daily and monthly scale

**Figure 10:**
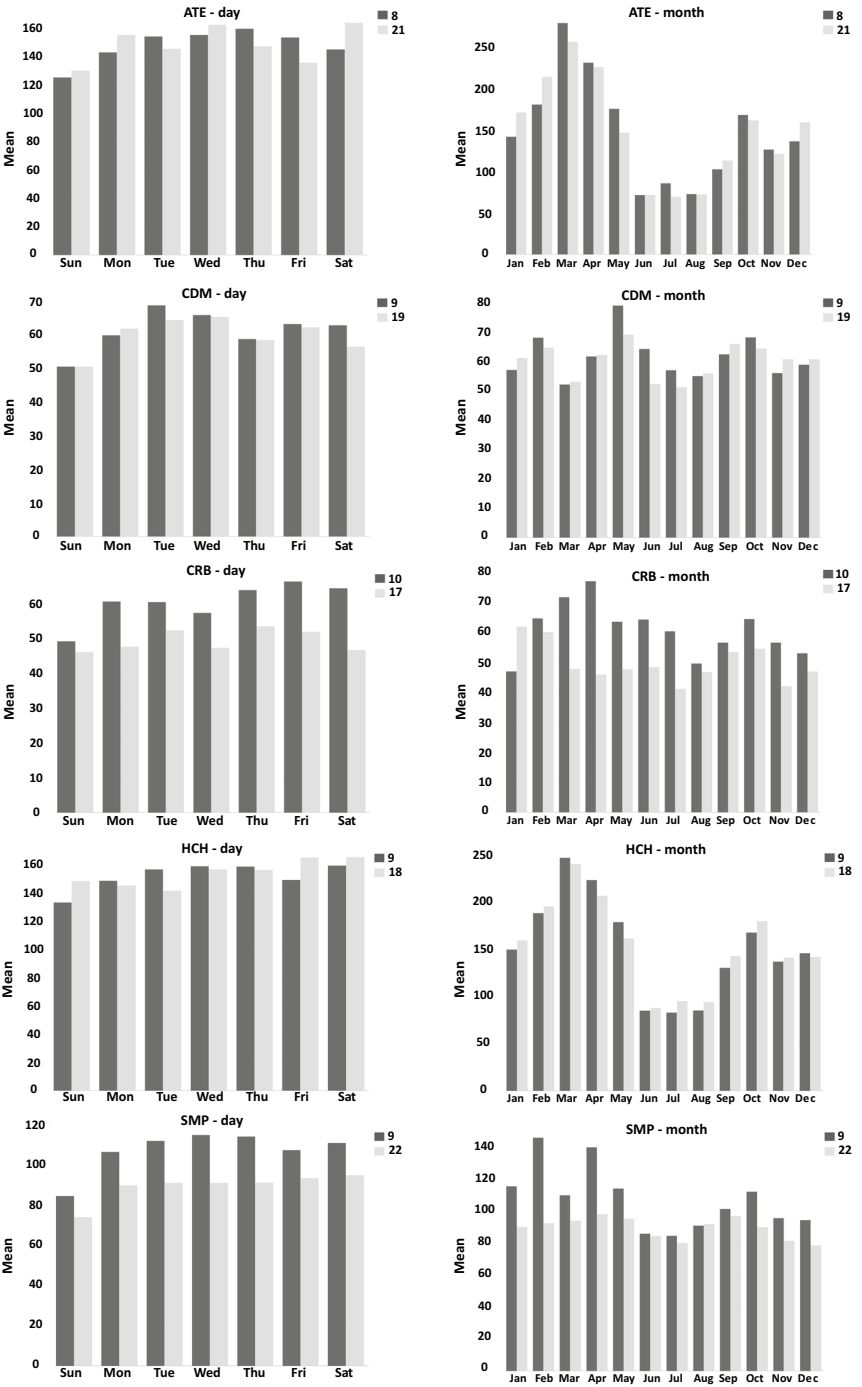
Bar plot per day and month for each monitoring station, ATE, CDM, CRB, HCH, and ATE, respectively. The average hourly pollution per day of the week and month of the year is reported for all monitoring stations.

The predominant time scale in the concentration of $\mathrm {PM_{10}}$ was evaluated in two episodes (see Fig. 10). That between 07:00 and 11:00 in the morning, followed by the one between 17:00 and 22:00 at night. Similar results were found by Sánchez et al.^27^, where the air quality of Lima was evaluated in 2015. From the above, it can be inferred that the levels of environmental pollution referring to $\mathrm {PM_{10}}$, find the highest peaks in the evening (153.9991 and $151.9256\, \mu \hbox {g}/\hbox {m}^{3}$), while the lowest peaks are between 03:00 and 04:00 a.m. each day, which coincides with the results reported for the station HCH. As mentioned by Valdivia et al.^3^, this is related to the reduction in emissions from mobile sources that are own of the dawn.

The behavior of concentration levels of contamination varies depending on the month. In each monitoring station, we can see two main peaks (see Fig. 10). The first corresponds to February, March, and April, which report the highest contamination in the first semester of the year. In this period, it is the beginning of classes for schoolchildren that intensifies vehicle activity. The end of the summer and the beginning of the autumn are the period associated with the time at which the thermal inversion occurs, which favors the generation of high peaks of $\hbox {PM}_{10}$ contamination^57^. The second peak involves the winter season and the beginning of spring, highlighting mainly October as part of the second semester of the year. Similar results were found by Encalada et al.^31^.

In these time windows, the stations with the highest critical episodes were HCH and ATE, while CRB had the lowest $\hbox {PM}_{10}$ concentrations. In addition, from the emissions of high traffic vehicular and fixed sources of pollution, the meteorological and topographic conditions of the study area cause the high emission of $\hbox {PM}_{10}$ in the air, exceeding the proposed standards in all cases by WHO.

### Air pollution forecasting results

In this study, we focus on the one-hour ahead prediction of the $\hbox {PM}_{10}$ concentration based on both the past values of the pollutant concentration and the current weather variables. For this, the MLP and LSTM were used with a particular architecture. Based on the autocorrelation function (ACF) and the partial autocorrelation function (PACF), relevant lags were detected that are used in the model. The configuration of the network is associated with the information provided by the ACF and PACF, where the lags $t-1$, $t-2$, $t-3$, $t-23$, and $t-24$ of the $\hbox {PM}_{10}$ time series are defined as relevant. In addition, temperature, relative humidity, and wind speed are used with $t-4$ (4 hours ago). In summary, the non-linear autoregressive model with exogenous variables identified has the following structure:

15 $$\begin{aligned} X_t = g_{ANN}(X_{t-1}, X_{t-2}, X_{t-3}, X_{t-23}, X_{t-24}, Temperature_{t-4}, Humidity_{t-4}, Wind_{t-4}) + \varepsilon _t \end{aligned}$$

where $\{X_t, t\in {\mathbb {N}}\}$ is the $\hbox {PM}_{10}$ time series. The weather exogenous variables are $\{Temperature_{t}, t\in {\mathbb {N}}\}$, $\{Humidity_{t}, t\in {\mathbb {N}}\}$ and $\{Wind_{t}, t\in {\mathbb {N}}\}$ for temperature, humidity and wind speed respectively. Moreover, $\varepsilon _t$ is the random noise. The non-linear function $g_{ANN}(\cdot )$ stands for either the MLP or the LSTM neural networks.

The purpose of incorporating exogenous variables in this study is to improve the precision of the forecast. The exogenous variables are crucial to improve the efficiency of predictions by identifying the important meteorological covariates that affect $\hbox {PM}_{10}$, such as temperature, relative humidity, and wind speed^72^.

In this work, we have implemented a three-layer MLP with 8 input nodes, 16 hidden nodes, and 1 output node. The activation function for the hidden and output nodes is the sigmoid function $f(z)=(1+e^{-z})^{-1}$. On the other hand, the LSTM was implemented with 16 parallel blocks, and the output of each block is aggregated with a single neuron with a sigmoid activation function. To train both ANN models, we have selected the mean absolute error for the loss function as a robust function due to outliers. The *nadam* optimizer was used for the backpropagation algorithm. A 25% dropout strategy with a 10% of validation data was applied to avoid over-fitting. A maximum of 500 epochs and batch sizes of 1024 was used to fit the models’ weights.

**Figure 11:**
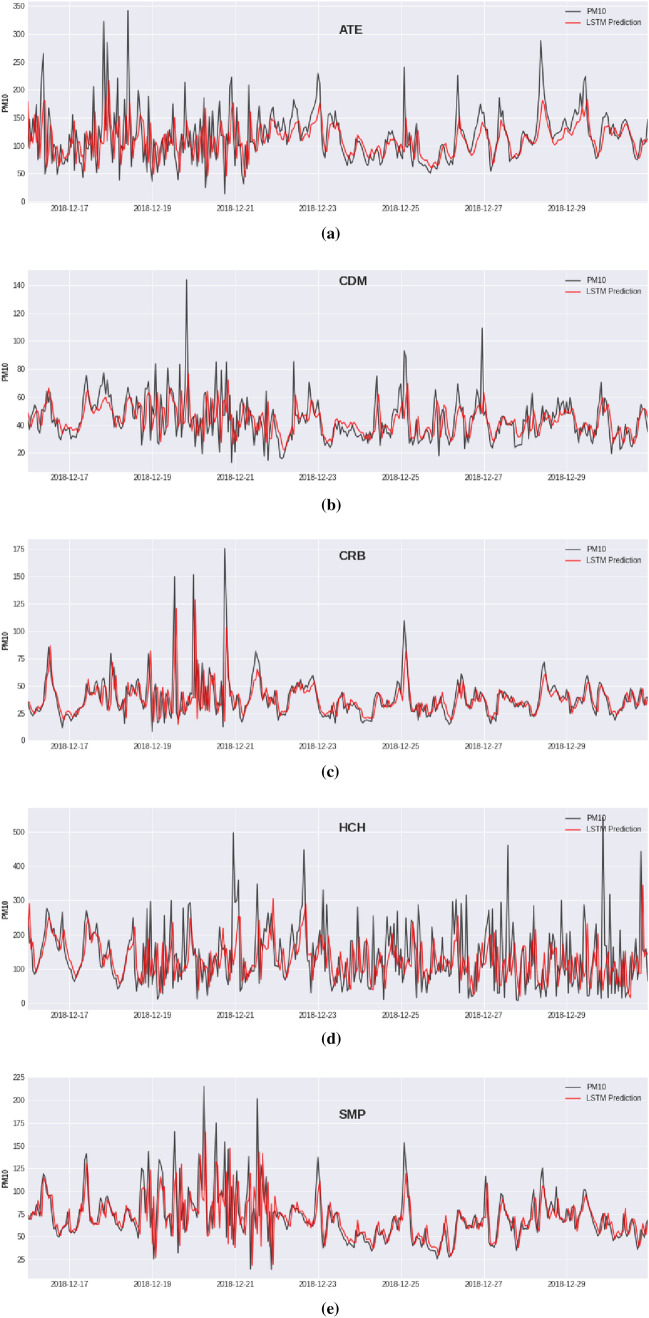
Plots for one-hour ahead predictions for the last 15 days of the $\hbox {PM}_{10}$ concentration level using LSTM with the BNCV scheme. Predictions for the following monitoring stations: (**a**) ATE, (**b**) CDM, (**c**) CRB, (**d**) HCH, (**e**) SMP.

Two alternatives were considered to obtain out-of-sample forecasts (see Fig. 11). On the one hand, the ANN models were adjusted with the training set only once for the Hold-Out scheme, and the resulting model was used to forecast one-hour ahead for the last 60 days of data. On the other hand, the ANN modes were trained several times with a fixed sliding window for the Blocked Nested Cross-Validation, where the model was updated for each subsequent day belonging to the test set, and the following days (24 samples) were used for the test set.

Table 3 shows the performance results obtained by the MLP and LSTM models evaluated in the test set using the Hold-Out and the Blocked Nested Cross-Validation Schemes. Figure 11 shows the graphs obtained by the predictions of the LSTM neural network for the five monitoring stations. Artificial neural networks show good prediction performance according to the Spearman score (over 0.60) for all the stations, except for ATE that reaches a score near 0.52. ATE and HCH monitoring stations are located in industrial areas with heavy traffic stations. The ATE and HCH monitoring stations have the highest levels of contamination and a more significant presence of outliers, which is reflected in the error metrics with values greater than twice that of the other stations. Notice that RMSE shows a higher value due to the presence of extreme values in the $\hbox {PM}_{10}$ levels, being MAE less affected by this type of value. On the other hand, the models evaluated by applying the BNCV scheme show slightly better performance than their HO counterparts. However, the BNCV scheme keeps the models updated with the latest records through an incremental training process with the new data.

The models’ performances were strongly affected by a period of excessive contamination with critical episodes that appeared between December 3rd, 2018, and December 21st, 2018 (just before the Christmas festivities).

**Table 3:** Performance results for the MLP and LSTM models were evaluated using The Hold-Out and the Blocked Nested Cross-Validation schemes. The summary of the results corresponds to one-hour ahead predictions of the concentration levels of the pollutant $\hbox {PM}_{10}$ evaluated in the last 60 days of the data set.

Metrics	ATE		CDM		CRB		HCH		SMP	
	MLP	LSTM	MLP	LSTM	MLP	LSTM	MLP	LSTM	MLP	LSTM
Hold-Out scheme										
MAE	27.458	27.637	9.639	9.609	6.577	6.548	42.740	41.514	10.441	10.105
RMSE	45.752	46.509	13.771	13.743	10.573	10.682	64.297	62.903	15.959	15.520
sMAPE	24.059	24.071	19.344	19.328	17.283	17.208	33.846	32.829	14.331	13.935
Spearman r	0.517	0.514	0.658	0.660	0.756	0.755	0.649	0.663	0.815	0.823
Blocked Nested Cross-Validation scheme										
MAE	26.845	27.066	9.689	9.562	6.644	6.339	44.586	43.191	10.155	9.696
RMSE	44.718	45.923	13.885	13.808	10.840	10.722	64.785	63.690	16.162	15.752
sMAPE	23.590	23.607	19.499	19.240	17.280	16.639	35.54	34.569	14.012	13.467
Spearman r	0.523	0.520	0.654	0.657	0.756	0.766	0.632	0.648	0.815	0.817

The time series of the pollutant was decomposed into trend, seasonality and irregular components using the decomposition method described in equation 2. The irregular component was subtracted from the original time series, and filtered time series is obtained:

16 $$\begin{aligned} {\tilde{X}}_t = Trend_t + Seasonal_t \end{aligned}$$

**Figure 12:**
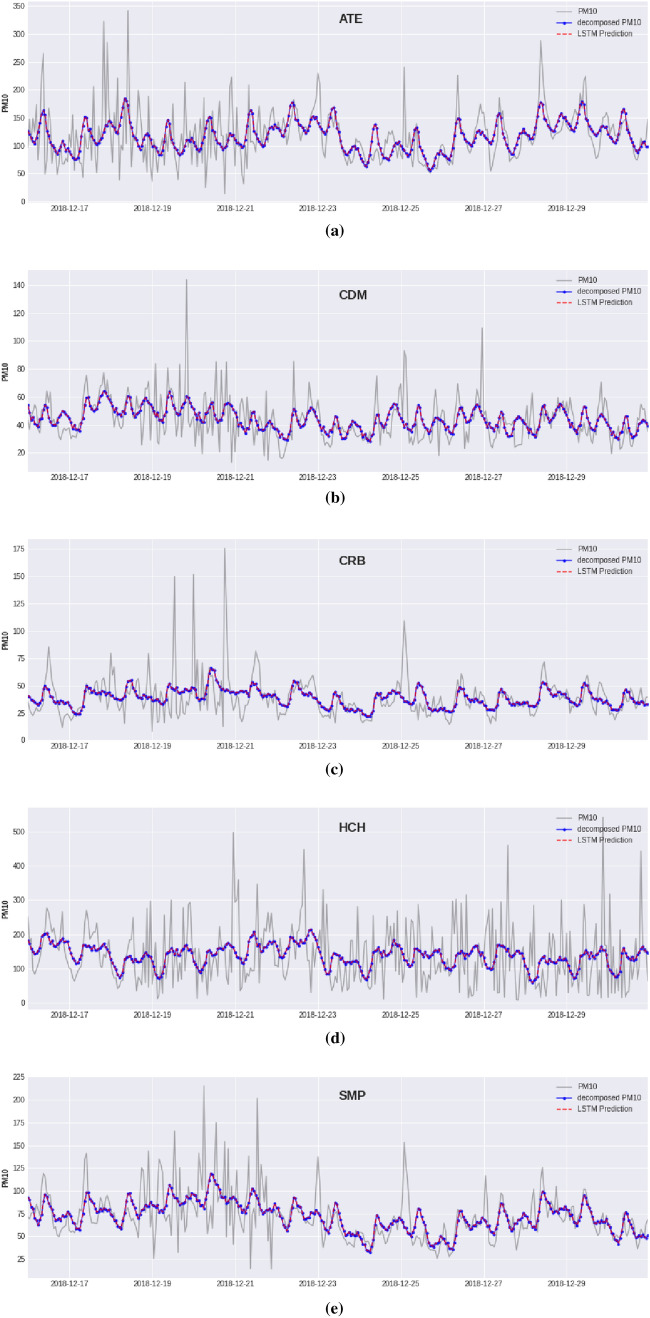
Plots for one-hour ahead predictions for the last 15 days of the regular component of the $\hbox {PM}_{10}$ concentration level using LSTM with the BNCV scheme. Predictions for the following monitoring stations: (**a**) ATE, (**b**) CDM, (**c**) CRB, (**d**) HCH, (**e**) SMP.

Table 4 shows the performance results obtained by the MLP and LSTM models evaluated in the test set using the Hold-Out and the Blocked Nested Cross-Validation Schemes applied to the filtered time series. Under this situation, both the MLP and the LSTM performed very well in predicting the regular component of the $\hbox {PM}_{10}$ contamination levels at all monitoring stations. A remarkable point is an outstanding performance obtained by the artificial neural network models, which shows that the irregular component is hard to predict. Figure 12 shows the graphs obtained by the predictions of the LSTM neural network for the five monitoring stations.

**Table 4:** Performance results for the MLP and LSTM models were evaluated using The Hold-Out and the Blocked Nested Cross-Validation schemes. The summary of the results corresponds to one-hour ahead predictions of the filtered time series of the concentration levels of the pollutant $\hbox {PM}_{10}$ evaluated in the last 60 days of the data set.

Metrics	ATE		CDM		CRB		HCH		SMP	
	MLP	LSTM	MLP	LSTM	MLP	LSTM	MLP	LSTM	MLP	LSTM
Hold-Out scheme										
MAE	4.203	2.659	1.737	1.336	1.628	1.423	6.370	4.255	2.830	1.941
RMSE	5.724	3.706	2.235	1.732	2.192	1.844	8.324	5.837	3.602	2.299
sMAPE	3.646	2.411	3.581	2.830	4.269	3.927	4.636	3.224	4.063	2.867
Spearman r	0.986	0.991	0.973	0.982	0.967	0.974	0.981	0.988	0.982	0.990
Blocked Nested Cross-Validation scheme										
MAE	4.217	2.720	1.829	1.325	1.645	1.333	6.621	4.561	2.749	1.841
RMSE	5.738	3.731	2.350	1.712	2.297	1.835	8.622	6.101	3.558	2.194
sMAPE	3.619	2.468	3.743	2.810	4.330	3.575	4.905	3.454	3.856	2.709
Spearman r	0.984	0.991	0.973	0.982	0.963	0.973	0.977	0.987	0.980	0.991

### Comparison of the present study with past studies

This section shows the comparison of the present study with other previous studies on the evaluation and prediction of $\hbox {PM}_{10}$ in Lima, showing the duration of the study and the main findings. It is observed that our results agree with the other studies in that vehicular traffic is the main activity that causes critical episodes of $\hbox {PM}_{10}$, and this is exacerbated in the summer months.

- Silva et al.^28^ shows that the highest concentrations of $\hbox {PM}_{10}$ were observed in the eastern part of the city. The main sources of particulate material are the large open areas, vehicular traffic, the commercialization of rubble, bricks, and cement. The highest concentrations of $\hbox {PM}_{10}$ are observed in summer. **Pollutant types**: $\hbox {PM}_{10}$, $\hbox {PM}_{2.5}$. **Duration of study**: 6 years (2010-2015).
- Reátegui-Romero at al.^73^ show that, for the monitoring stations in the eastern zone, the highest concentrations of $\hbox {PM}_{10}$ are observed in the northern area of Lima, the Relative Humidity is inversely proportional to the concentrations of $\hbox {PM}_{10}$, higher peaks are observed in the summer month. **Pollutant types**: $\hbox {PM}_{10}$, $\hbox {PM}_{2.5}$. **Duration of study**: 2 months (February and July 2016).
- Sanchez et al.^10^ show that there is a higher concentration of $\hbox {PM}_{10}$ in the areas with the greatest impact of vehicular traffic, reaching maximum concentrations of 476,8 $\mu$g/$\hbox {m}^{3}$ for Santa Anita station. They used the WRF-Chem model to predict $\hbox {PM}_{10}$ concentrations, obtaining low precision results. **Pollutant types**: $\hbox {PM}_{10}$. **Duration of study**: 33 days (2016).
- In our study, we have specified that the major sources of the pollutant $\hbox {PM}_{10}$ are the vehicle fleet, the industrial park, and overcrowding, reaching maximum peaks of 974 $\mu$g/$\hbox {m}^{3}$ at the HCH station. The highest concentrations were observed in the summer months. Artificial neural networks were used, specifically, the LSTM model under two validation schemes to predict $\hbox {PM}_{10}$ concentrations. The results showed good prediction performance for both low concentrations and critical episodes. **Pollutant types**: $\hbox {PM}_{10}$. **Duration of study**: 2 years (2017-2018).

## Limitations

This study has some limitations. First, the number of data points represents a relatively short period (two years). A more extended period of hourly data may have allowed a more rigorous statistical analysis and more conclusive results. It is worth mentioning that the data related to $\hbox {PM}_{10}$ in Lima requires greater attention since many stations do not have the pertinent record of this pollutant, added to the scarce existing research related to this topic. Second, the collection of data related to other meteorological variables was also restricted since the monitoring stations do not record correctly for the most part. Third, the study does not consider data related to vehicular traffic or hospital care; the use of both variables may have enriched the research. However, our findings from the $\hbox {PM}_{10}$ analysis are consistent and complementary to a recent study showing the visual and exploratory aspect of the pollutant^31^. In addition, the MLP and LSTM architectures that allowed the analysis of predictions under two validation schemes are the precedent for future work with a predictive approach, being the first study in Lima that addresses the prediction of $\hbox {PM}_{10}$ using neural networks artificial. Likewise, it will be a support in the taking of preventive actions to critical environmental episodes.

## Conclusions

This study addressed the problem of forecasting $\hbox {PM}_{10}$ concentration on an hourly scale based on air quality indicators from five monitoring stations in Lima, Peru. A comparative study was accomplished between the MLP and LSTM neural networks evaluated with the Hold-Out and Blocked Nested Cross-Validation.

The MLP and LSTM can use the data from the previous period to accurately forecast the value of the $\hbox {PM}_{10}$ concentration in a short time ahead. They can learn the $\hbox {PM}_{10}$ concentration trends accurately. However, the performance is diminished when a station is subject to unpredictable external sources of pollution or due to short-term changes in climate and landforms (ATE and HCH). In this sense, the LSTM with the BNCV could better adapt to data from the monitoring stations that present episodes of extreme values. The results show that periods of moderate $\hbox {PM}_{10}$ concentration are predicted with very high precision. While for periods of high contamination, the model’s accuracy is diminished, although in any case, it has a reasonable degree of predictability.

Using a high-performance model in air quality forecasting in large cities, such as Lima, can help develop critical health protection and prevention tools. Deep learning neural networks such as the LSTM are crucial in helping design public policies that prioritize improving air quality conditions to develop more sustainable cities.

The different configurations of the LSTM respond to the forecast of $\hbox {PM}_{10}$ events by selecting the relevant meteorological variables. Precisely, the essential property of the LSTM is that through its memory units, they can remember the patterns over time, which is beneficial when forecasting $\hbox {PM}_{10}$. In this sense, LSTM with BNCV could better adapt to data from the monitoring stations that present episodes of extreme values.

The results show that the $\hbox {PM}_{10}$ concentration prediction achieves better results with artificial intelligence methods since they are suitable for this type of approach. However, it is proposed to conduct this type of study with other cross-validation methods and hybrid and ensemble methods, giving greater precision in the prediction. This study will help in decision-making regarding air pollution mitigation and strategies, not only in Lima but also in other cities in the country and abroad. In this sense, this study of $\hbox {PM}_{10}$ could be extrapolated to other pollutants, both at a national and international level. In fact, a recent study^74^ showed that genetic programming had higher prediction accuracy than artificial neural networks and was equally competent for peak predictions. Further works are required to explore other methods (hybrid or ensemble) to increase the accuracy of predictions.

As future work, we expect to apply other variants of deep learning models that include incremental learning^75^, as well as to introduce self-identification techniques for the model identification^41,76^.
